# Supplementary material for: Optimal minimal residual disease threshold in pediatric acute myeloid leukemia: A retrospective cohort study based on the TARGET database
Source: PLoS Med. 2026 May 8;23(5):e1005088. doi: 10.1371/journal.pmed.1005088 (PMC13155632; doi:10.1371/journal.pmed.1005088)
Supplement: S1 Code — (ZIP) [file pmed.1005088.s002.zip › S2 code/PROJ8_4_tbl1/PROJ8_4_tbl1_seg1_0.pdf]

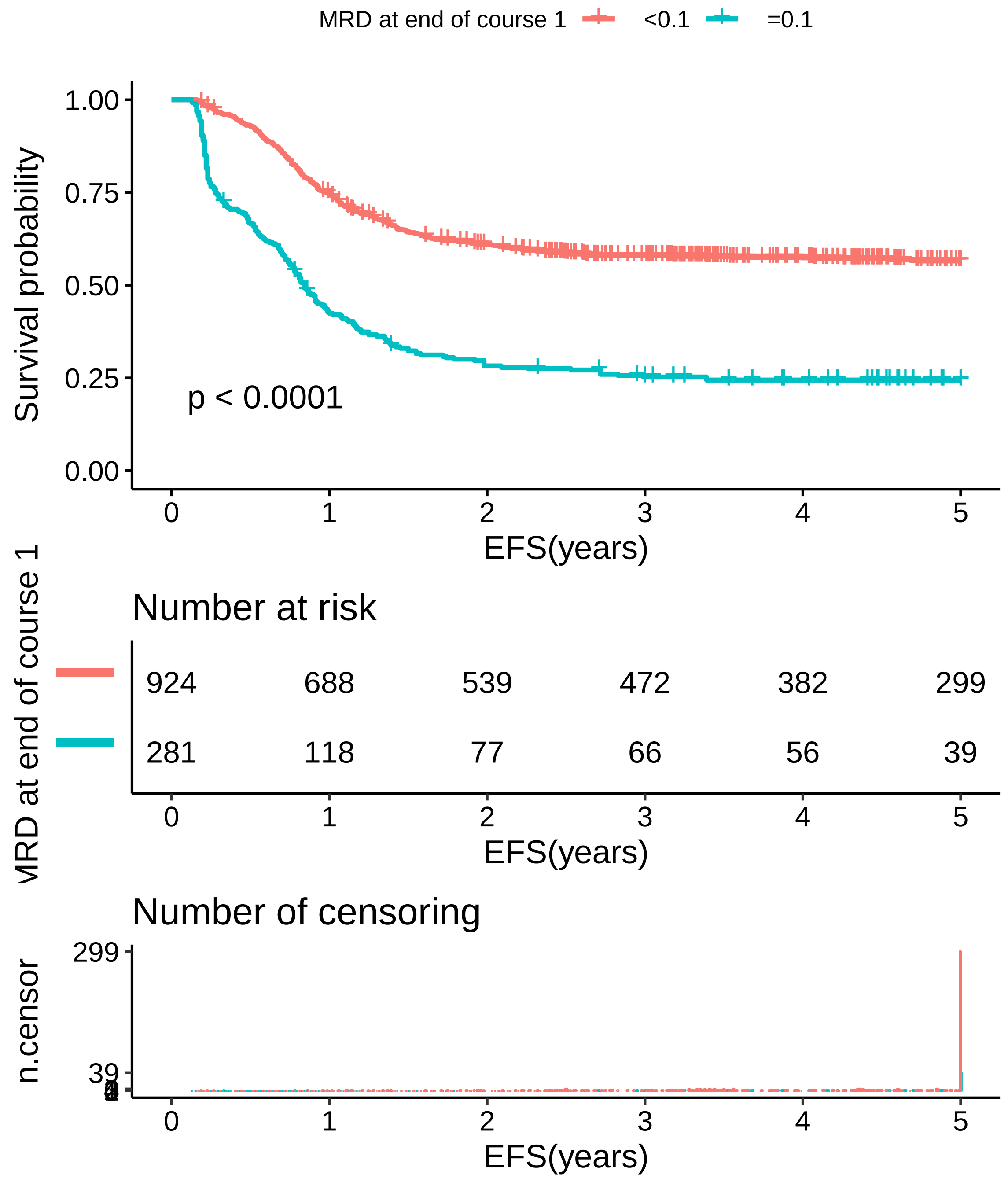

MRD at end of course 1
<0.1
=0.1
Survival probability
1.00
0.75
0.50
0.25
0.00
p < 0.0001
0
1
2
3
4
5
EFS(years)
Number at risk
MRD at end of course 1
924 688 539 472 382 299
281 118 77 66 56 39
0
1
2
3
4
5
EFS(years)
Number of censoring
n.censor
299
39
0
1
2
3
4
5
EFS(years)
